# Identification of Hypotensive Biofunctional Compounds of *Coriandrum sativum* and Evaluation of Their Angiotensin-Converting Enzyme (ACE) Inhibition Potential

**DOI:** 10.1155/2018/4643736

**Published:** 2018-11-15

**Authors:** Farieha Hussain, Nazish Jahan, Khalil-ur- Rahman, Bushra Sultana, Saba Jamil

**Affiliations:** ^1^Department of Chemistry, University of Agriculture, Faisalabad 38000, Pakistan; ^2^Department of Biochemistry, University of Agriculture, Faisalabad 38000, Pakistan

## Abstract

The aim of this study was to identify and characterize the bioactive compounds of *Coriandrum sativum* responsible for the treatment of hypertension and to explore their mechanism of action as angiotensin-converting enzyme (ACE) inhibitors. Bioactive fractions like alkaloids, flavonoids, steroids, and tannins were extracted and evaluated for their ACE inhibition potential. Among them, only flavonoid-rich fraction showed high ACE inhibition potential with IC_50_ value of 28.91 ± 13.42 *μ*g/mL. The flavonoids were characterized through LC-ESI-MS/MS. Seventeen flavonoids were identified in this fraction of *Coriandrum sativum* in negative ionization mode which includes pinocembrin, apigenin, pseudobaptigenin, galangin-5-methyl ether, quercetin, baicalein trimethyl ether, kaempferol dimethyl ether, pinobanksin-5-methylether-3-O-acetate, pinobanksin-3-O-pentenoate, pinobanksin-3-O-phenylpropionate, pinobanksin-3-O-pentanoate, apigenin-7-O-glucuronoide, quercetin-3-O-glucoside, apigenin-3-O-rutinoside, rutin, isorhamnetin-3-O-rutinoside, and quercetin dimethyl ether-3-O-rutinoside, while six flavonoids including daidzein, luteolin, pectolinarigenin, apigenin-C-glucoside, kaempferol-3-7-dimethyl ether-3-O-glucoside, and apigenin-7-O-(6-methyl-beta-D-glucoside) were identified in positive ionization mode. The results of this study revealed that *Coriandrum sativum* is a valuable functional food that possesses a number of therapeutic flavonoids with ACE inhibition potential that can manage blood pressure very efficiently.

## 1. Introduction

Hypertension is a dominant risk factor for chronic diseases, including cardiovascular disorders, stroke, renal diseases, and diabetes. Hypertension is the second leading cause of disability around the world, and its prevalence has doubled in the last 5 years in all social strata of Pakistan. Globally, the overall prevalence of hypertension is approximately 40% in adults. Among them, almost 57% of human papulation is well aware about their blood pressure, from which about 40.6% have access to antihypertensive medicines. Still, there is a huge gap between the number of hypertensive patients and the availability of treatment facilities. The health facilities for the management of hypertension is more alarming in the middle and low income countries as more than 80% of deaths due to cardiovascular diseases occur in these countries [1]. The prevalence of hypertension in Pakistan is very high and is considered as a top alarming physiological disorder in the community [2]. Not to speak of Pakistan, hypertension has become a health menace all over the world. Owing to the high prevalence and increased mortality and morbidity due to hypertension and associated complications, there is a dire need to explore the alternatives including food components for the management of this health menace.

Bioactive phytoconstituents, available as natural components in foods and medicinal plants, provide preventive and curative health benefits to improve cardiovascular health. Functionalities of bioactives from green resources including inhibition of activity of enzymes or form complexes with metals, which catalyse the oxidation reaction and the capacity to modulate metabolic processes, may result in the eradication and management of cardiovascular diseases [3]. Angiotensin I-converting enzyme (ACE) plays a fundamental role in the management of hypertension as it coverts angiotensin I to the potent angiotensin II which is vasoconstrictor. Moreover, it inactivates the vasodilator bradykinin. By inhibiting these processes, synthetic ACE inhibitors have long been used as antihypertensive agents. However, the unwanted side effects of these drugs are taking their toll in the form of side effects with high rate. Bioactive food components like alkaloids, peptides flavonoids, flavanols, anthocyanins, phenolic acids, polyphenols, tannins, resveratrol, polysaccharides, and sterol have been identified as green ACE inhibitors [4]. These nutritional components have received considerable attention for their effectiveness in both the prevention and the treatment of hypertension. The recent research emphasis is on the characterization of the bioactive constituent of foods and identification of their molecular mechanism of action for the development of drugs from green natural resources. Flavonoids are reported to exhibit the capacity to inhibit different zinc metalloproteinases, including ACE. Indeed, the micromolar concentrations of various flavonoids, such as anthocyanins, flavones, flavonols, and flavanols, have been reported to exhibit more than 50% of ACE inhibition potential. Therefore, different bioactive flavonoids, including catechin, epicatechin, rutin, myricetin, luteolin, apigenin, and naringenin, were identified from the leaves of *Mentha spicata* [5]. 2-(3,4-dihydroxy-5-methoxy-phenyl) and 3,5-dihydroxy-6,7-dimethoxychromen-4-one were extracted from the leaves of *Euphorbia neriifolia*. Epicatechin, (-)-(2S)-6-Methoxy-[2^″^,3^″^: 7,8]-furanoflavanone, kaempferol-3-O-sulphate-7-O-c-arabinopyranoside, vidalenolone, (2S)-7, 8, bi's-3′, 4′-(2,2-dimethyl-chromano)-5-hydroxy flavanone, 3,7-dihydroxy-4′,8-dimethoxy flavone, 14-Hydroxy artonin E, and kaempferol were extracted from the leaves and flowers of *Cassia angustifolia* [6]. Rutin and kaempferol were also extracted from *Ficus carica* and evaluated for their therapeutic potential [4, 7, 8]. Many flavonoids of *Coriander sativum* with known ACE inhibition mode of action were explored for the first time in this study.

*Coriandrum sativum L*. belonged to the family Apiaceae (Umbelliferae) and mainly cultivated throughout the year. It is an important functional food that is traditionally used as nutrition and taste enhancer with additional medicinal benefits including antioxidant, antihypertensive, lipid lowering, and analgesic [9]. *Coriandrum sativum*, being a treasure of bioactive compounds, may contribute its exceptional pharmaceutical potential to combat cardiovascular diseases [10]. Therefore, *Coriandrum sativum* was explored for the identification and characterization of more bioactive compounds with known ACE inhibition mechanism of action against hypertension.

## 2. Materials and Method

All analytical grade chemicals and reagents were purchased from Sigma-Aldrich, USA, and Merck, Germany. *Coriandrum sativum* (fresh leaves) was purchased from the local market and identified from a taxonomist of Department of Botany, University of Agriculture Faisalabad (voucher no. 227-4-2016).

### 2.1. Extraction of Bioactive Fractions of Phytochemicals

Four bioactive fractions of phytoconstituents were extracted from *Coriandrum sativum.*

#### 2.1.1. Extraction of Flavonoids

The extraction of partially purified flavonoid fraction was made through Soxhlet apparatus by using multiple solvents from least to most polar with the order of n-hexane, chloroform, ethyl acetate, and methanol (500 mL of each). Each fraction was examined for the presence of flavonoid with the appearance of orange-pink color through Shibata reaction [11, 12]. Flavonoid positive ethyl acetate fraction was dried by evaporating solvent through rotary evaporator (BUCHI Rotavapor II) at 50°C to obtain brownish mass. About 1 mg of brownish mass was dissolved in 10 mL of distilled water and used for the evaluation of ACE inhibition potential of flavonoids.

#### 2.1.2. Extraction of Tannins

Defatted plant material was shaked for four days at ambient temperature with mixture of acetone and water (70 : 30 *V*/*V*) in a capped flask. Acetone was evaporated by using rotary evaporator, and remaining aqueous extract was further partitioned with dichloromethane (2 × 50 mL) and diethyl acetate (4 × 50 mL) separately. The organic layers were dried by evaporating the solvent under reduced pressure at 50°C, and the presence of tannins were confirmed with the formation of greenish-black precipitate by adding 5% ferric chloride [11].

#### 2.1.3. Extraction of Alkaloids

Dried powder of *Coriandrum sativum* (20 g) was extracted with benzene for 6 hours. This benzene extract was shaked with three successive portions of 5% sulphuric acid (25 cm^3^) and decolorized with activated charcoal. The hot solution was filtered. The pH of the filtrate was maintained at 8.5 with ammonia solution. Filtrate was transferred into a separatory funnel and extracted with three successive portions of chloroform (20 cm^3^). All three portions were combined, and chloroform layer was distilled off to get alkaloids. The presence of alkaloids was confirmed by the appearance of reddish precipitate with Dragendorff's reagent [11].

#### 2.1.4. Extraction of Steroids

The grinded plant material (40 g) was soaked in 200 mL of ethanol for 7 days, and the gummy material was obtained after ethanol evaporation under reduced pressure at 40°C by using rotary evaporator. This gummy material was redissolved in 90% ethanol and partitioned successively with n-hexane, chloroform, and ethyl acetate through Soxhlet apparatus. All three fractions were tested for presence of steroids by adding acetic anhydride and H_2_SO_4_ [13]. The ethyl acetate fraction showed positive results for presence of steroid.

### 2.2. Evaluation of ACE Inhibition Potential

#### 2.2.1. Preparation of Lung Acetone Powder

The lungs, separated from freshly slaughtered rabbits, were washed with 0.8% saline solution and centrifuged at 4000 rpm for 10 minutes with phosphate saline buffer. The supernatant was removed, and the residue was washed with acetone with continuous stirring on a magnetic stirrer. After overnight drying, this material was grinded to fine powder and stored at 4°C as lung acetone powder [14].

#### 2.2.2. Extraction of ACE from Lung Acetone Powder

The extraction of enzyme was carried out by mixing lung acetone powder (0.5 g) and 10 mL of borate buffer (100 mM, pH 8.3) with continuous overnight stirring. Then this mixture was centrifuged at 4000 rpm for 45 minutes. Supernatant was dialyzed with borate buffer by using dialyzing membrane (pore size 20 A°). The ACE enzyme, collected after lyophilization, was stored at −20°C [15].

#### 2.2.3. ACE Activity Assay

ACE activity was determined by the following method of Belovic et al. [16] with some modifications. The solution of angiotensin-converting enzyme (50 *μ*L of 100 mU/mL) was incubated with 50 *μ*L borate buffer at 37°C for 10 minutes and was reincubated for 80 minutes at 37°C after adding substrate, Hip-His-Leu (150 *μ*L, 8.3 mM in borate buffer). The reaction was stopped with 250 *μ*L of 1 M HCl. The resulting hippuric acid was extracted with 1500 *μ*L of ethyl acetate after centrifugation at 3000 rpm for 15 minutes. Supernatant (750 *μ*L) was dried under air flow at 7°C. In this dried powder, 1 mL of distilled water was mixed, and the absorption of hippuric acid released after action of ACE was measured at 228 nm by using UV/Visible double beam spectrophotometer. The reaction blank was prepared with the same procedure except the addition of HCl before adding substrate.

#### 2.2.4. ACE Inhibition Potential of Partially Purified Bioactive Fractions

The inhibition percentages were determined by the same modified enzyme assays only by replacing 50 *μ*L of buffer with the same volume and concentration of partially purified bioactive fractions (100 *μ*g/mL) and standard captopril (100 *μ*g/mL). The decrease in concentration of hippuric acid in test sample as compared to control was expressed in terms of percentage ACE inhibition.

ACE inhibition was calculated according to the following equation: %IACE = 100[(A − B) − (C − D)]/(A − B), where A represents absorbance in the presence of ACE, B absorbance of reaction blank, C absorbance in the presence of ACE and inhibitors, and D absorbance of sample blank.

### 2.3. LC-ESI-MS/MS Analysis of Secondary Metabolites

Secondary metabolites of *Coriandrum sativum* with the highest ACE inhibition activity were subjected to chemical characterization by LC-ESI-MS/MS analysis to find out the bioactive compounds actually involved in ACE inhibition potential. This analysis was carried out on liquid chromatography coupled with electrospray ionization (Linear Ion Trap, LTQ XL) mass spectrometer (ESI-LC/MS) (Thermo Fisher Scientific, San Jose, CA, USA).

In order to get chromatographic separations, 5 *μ*L of each sample was injected via auto sampler (Surveyor Autosampler Plus) into the HPLC system (Surveyor) equipped with Luna reverse phase C-18 column (250× 4.6 mm, 5 *μ*m particle size) (Phenomenex, USA). The elution of sample from column was carried out at a flow rate of 0.5 mL/min using gradient elution.

Solvent A and B were prepared by mixing water, acetonitrile, and trifluoroacetic acid (TFA) with a ratio of 90 : 10 : 0.1% (*v*/*v*) and 10 : 90 : 0.06% (*v*/*v*), respectively, for positive ionization mode. Solvent A and B were prepared by mixing water, acetonitrile, and formic acid with a ratio of 90 : 10 : 0.1% (*v*/*v*) and 10 : 90 : 0.06% (*v*/*v*), respectively, for negative ionization mode. Gradient elution was programmed as follows: from 10% to 25% A and 90 to 75% B from 0 to 05 min followed by 25 to 50% A and 75 to 50% B in the next 10 min. This flow was maintained till the end of analysis. A photodiode array was used as the detector, and prominent peaks were further analyzed by mass spectrometer. The compounds corresponding to these peaks were ionized using atmospheric pressure electrospray ionization (ESI) probe at negative and positive ionization mode. ESI MS/MS conditions were selected as sheath gas flow rate 45 (arb) or 9 liter/min, auxiliary gas flow rate 10 (arb) or 2 liter/min. APCI vaporization temperature was maintained at 300°C, corona source voltage 4.5 KV, source current 4.10 *μ*A, ion transfer capillary temperature 275°C, capillary voltage 45 V, and tube lens voltage110 V.

The identification of flavonoids was performed under full scan mode at different ranges of *m/z*. MS^2^ analysis for each parent ion peak at different collision-induced dissociation (CID) powers. Xcalibur 1.4 software was applied for calibration of MS data [17–21].

Qualitative analysis was performed by comparing the retention time of identical peaks in LC-ESI-MS/MS chromatogram of present analysis with those of reference standards, literature reports, and library. The chemical nature of the compounds was proposed on the basis of MS and MS^2^ analysis fragmentation data. The fragmentation pattern gave tentative confirmation about the presence of a compound.

### 2.4. Statistical Analysis

The results were presented as mean ± SE of three concordant readings. The means were analyzed by one-way ANOVA followed by Tukey's test. IC_50_ values for % ACE inhibition of different extracts were calculated using linear regression analysis.

## 3. Results

### 3.1. Extraction of Bioactive Fractions and Evaluation of Their ACE Inhibition Potential

Four bioactive fractions including flavonoids, tannins, alkaloids, and steroids were extracted from *Coriandrum sativum* and evaluated for ACE inhibition potential, and the results have been presented in Table 1. Flavonoid fraction of *Coriandrum sativum* showed 81.4 ± 0.48% inhibition of ACE, while the potential of tannin fraction (2.3 ± 0.64%) to inhibit the ACE was very small. Alkaloid and steroid fractions of *Coriandrum sativum* have not revealed any ACE inhibitory activity. This high ACE inhibition potential of flavonoid fraction of *Coriandrum sativum* certifies that no other phytoconstituents including tannins, alkaloids, and steroids but only the flavonoids of this plant may manage hypertension through ACE inhibition mode of action. The studies reported the antihypertensive potential of tannins and alkaloids of some other plants may be due to the mode of action other than ACE inhibition, like calcium channel blocker or beta blockers [22].

**Figure 1:**
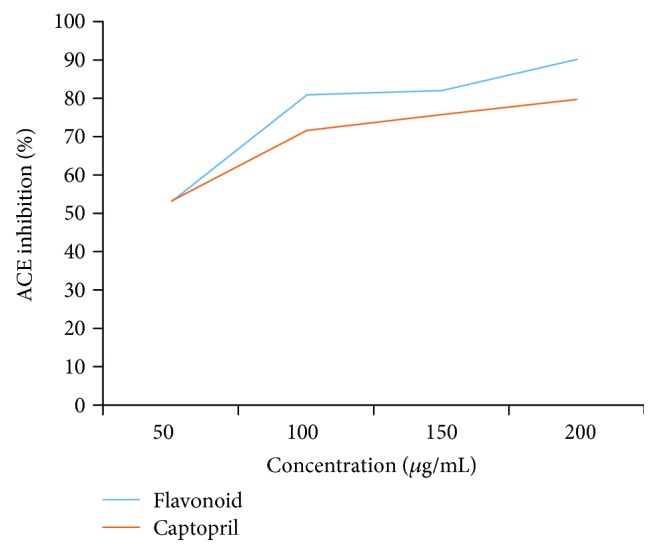
ACE inhibition (%) of flavonoids fraction of *Coriandrum sativum* and captopril at different concentrations.

Therefore, only the flavonoid fraction was further evaluated at its various concentrations to find its dose-dependent response and IC*_50_* of ACE inhibition. Standard synthetic drug captopril was also evaluated for ACE inhibition at different concentrations (Figure 1). The results revealed that the ACE inhibition potential of flavonoid fractions was increased with the increase of its concentration. The partially purified fraction of flavonoid and captopril exhibited almost equal ACE inhibition potential at 50 *μ*g/mL, but flavonoids depicted higher ACE inhibition potential at all other studied concentrations than the standard synthetic drug.

As for as IC_50_ values are concerned, the studied flavonoid-rich fraction of *Coriandrum sativum* showed higher IC_50_ value as compared to the captopril (Table 2).

### 3.2. LC-ESI-MS/MS Analysis of Flavonoid Fraction of *Coriandrum sativum*

In order to identify the specific compounds, the flavonoid fraction of *Coriandrum sativum* was further characterized through LC-ESI-MS/MS in both negative and positive ionization mode. Identification of the flavonoids was confirmed by comparing retention times and mass spectra with authentic standards available in the literature. In case of unavailability of any standards, the compounds were identified on the basis of accurate mass data of [M−H]^−^ and [M + H]^+^ ions.

**Figure 2:**
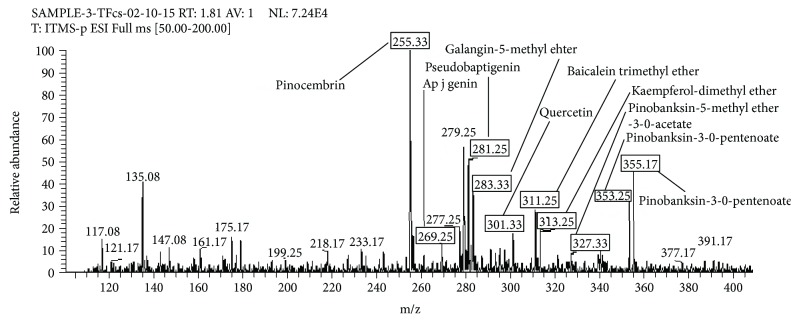
LC-MS/MS spectrum (*m/z* 120–400) of flavonoid fraction of *Coriandrum sativum* generated through negative ionization mode.

**Figure 3:**
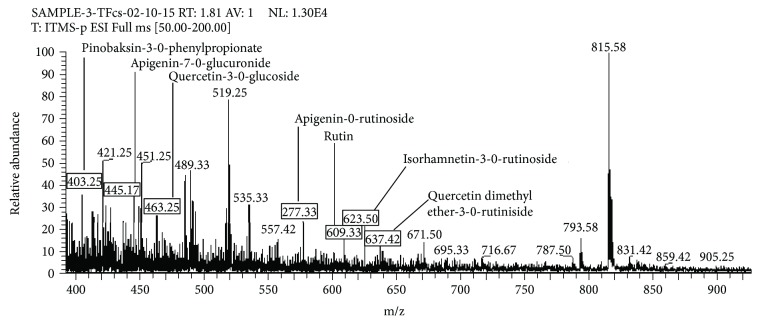
LC-MS/MS spectrum (*m/z* 400–900) of flavonoid fraction of *Coriandrum sativum* generated through negative ionization mode.

Seventeen flavonoid compounds were identified in negative ionization mode of LC-ESI-MS/MS (Figures 2 and 3). MS spectrum showed peak at 255.33 [M-H]^−^. This peak was identified as pinocembrin (C_15_H_12_O_4_) (Figure 2), which showed MS/MS fragment ion peaks at *m/z* 237.17 with the elimination of [M-H-OH]^−^, at *m/z* 213.17 after elimination of [M-H-C_2_H_2_O^−^]^−^, and at *m/z* 211.17 with the removal of [M-H-CO_2_]^−^ from the molecular ion (Table 3). Similar fragment ion peaks for pinocembrin have also been reported by Falcão et al. [23] and Falcão et al. [24] in the earlier literature.

A molecular ion peak at *m/z* 269.25 was identified as apigenin (C_15_H_10_O_5_) in mass spectrum (Figure 2). Apigenin produced fragment ion peaks in the MS^2^ spectrum at *m/z* 153.08 correspond to the removal of [M-H-116 amu]^−^ and at *m/z* 119.17 attributed to the loss of [M-H-150 amu]^−^ (Table 3). The fragment ions identified for apigenin in this study are in good harmony with fragment ions observed by Aldini et al. [25] in another study.

Peak at *m/z* 281.25 [M-H]^−^ (C_16_H_10_O_5_) in mass spectrum was proposed as pseudobaptigenin. MS/MS spectrum showed a fragment ion peak at *m/z* 263.25 that corresponds to the loss of a water molecule and at *m/z* 237.17 produced by the loss of CO_2_.

The precursor ion showed peak at *m/z* 283.33 [M-H]^−^ (C_16_H_12_O_5_) identified as galangin-5-methylether [M-H]^−^ (C_16_H_12_O_5_) in mass spectrum (Figure 2). MS/MS spectrum showed fragment ion peaks at *m/z* 268.08 by the loss of methyl radical [M-H-^·^CH_3_]^−^, at *m/z* 239.25 that corresponds to the loss of [M-H-CO_2_]^−^, and at *m/z* 211.17 by the elimination of [M-H-72]^−^ (Table 3). These fragment ion peaks are in good agreement with previously identified peaks by Pellati et al. [26].

Peak at *m/z* 301.33 in mass spectrum (Figure 2) was identified as quercetin [M-H]^−^ (C_15_H_10_O_7_). Quercetin was further confirmed through its specific fragmentation pattern in the MS/MS spectrum. The MS/MS spectrum of quercetin showed fragment ion peaks at *m/z* 273 by the loss of [M-H-CO]^−^, at *m/z* 257.25 with the elimination of [M-H-CO_2_]^−^, and at *m/z* 151.17 found after the elimination of [M-H-150 amu]^−^ (Table 3).

Peak at *m/z* 311.25 [M-H]^−^ (C_18_H_16_O_5_) in mass spectrum (Figure 2) was identified as baicalein trimethyl ether, which showed fragment ion peaks in the MS/MS spectrum at *m/z* 257.17 that corresponds to the loss of [M-H-CO_2_]^−^, at *m/z* 249.25 due to the loss of [M-H-CH_3_OH]^−^, and at *m/z* 153.00 by the loss of [M-H-B&C ring]^−^ (Table 3).

Peak at *m/z* 313.75 in mass spectrum (Figure 2) was identified as kaempferol dimethyl ether [M-H]^−^ (C_17_H_14_O_6_), which showed MS^2^ fragment ion peaks at *m/z* 298.08 with the elimination of [M-H-^·^CH_3_]^−^ (Table 3). Similar fragment ion peaks for kaempferol dimethyl ether were also previously reported by Falcão et al. [23] and Falcão et al. [24].

Peak related to *m/z* 327.33 [M-H]^−^ (C_18_H_16_O_6_) in mass spectrum (Figure 2) was identified as pinobanksin-5-methyl ether-3-O-acetate, which was further confirmed by MS^2^ fragment ion peaks at *m/z* 285.33 [M-H-CH_3_CHO^−^], at *m/z* 267.08 that corresponds to the loss of [M-H-CH_3_COOH]^−^, and at *m/z* 239.25 that corresponds to the loss of [M-H-CH_3_COOH-CO]^−^ (Table 3), as peaks identified for pinobanksin-5-methyl ether-3-O-acetate is in good agreement with the peaks described by Gardana et al. [27] and Falcão et al. [24] in previous data.

In the mass spectrum, peak that corresponds to *m/z* 353.25 [M-H]^−^ (C_20_H_18_O_6_) was identified as pinobanksin-3-O-pentenoate. The molecular ion peak of pinobanksin-3-O-pentenoate was confirmed through MS/MS fragment ion peaks at *m/z* 271.33 by the loss of [M-H-pentenal] and at *m/z* 253.08 by the loss of [M-H-pentenioc acid]^−^ (Table 3). These peaks are in harmony with peaks previously mentioned by Falcão et al. [28] and Falcão et al. [29].

Peak at *m/z* 355.17 [M-H]^−^ (C_20_H_20_O_6_) in the same mass spectrum was identified as pinobanksin-3-O-pentanoate, which was further confirmed by MS/MS fragment ion peak at *m/z* 271.33 by the loss of [M-H-pentanal]^−^ and at *m/z* 253.08 by the loss of [M-H-pentanioc acid]^−^ (Table 3). This fragmentation pattern showed harmony with earlier reported data by Falcão et al. [23] and Falcão et al. [24].

Peak at *m/z* 403.25 [M-H]^−^ (C_24_H_20_O_6_) in mass spectrum (Figure 3) was identified as pinobanksin-3-O-phenyl propionate. The molecular ion peak exhibited MS/MS fragment ion peaks at *m/z* 271.25 by the loss of [M-H- phenyl propanol]^−^ and at *m/z* 253.17 by the loss of [M-H-phenyl propanoic acid]^−^ (Table 3). The similar pattern of fragments ions also described in some other studies reported by Gardana et al. [27] and Falcão et al. [23].

A precursor ion at *m/z* 445.17 [M-H]^−^ (C_21_H_18_O_11_) in mass spectrum (Figure 3) was identified as apigenin-7-O-glucuronide. The MS/MS spectrum of this ion showed a product ion peak at *m/z* 269.08 that was corresponding to the loss of [M-H-glucuronide]^−^ and product ion at *m/z* 175.17 that corresponds to the loss of [M-H-glucuronide-C ring]^−^ (Table 3). Jeyadevi et al. [30] was also reported the fragment ion peaks at *m/z* 269 and *m/z* 175 for apigenin-7-O-glucuronide.

The same mass spectrum (Figure 3) showed that a precursor ion peak at *m/z* 463.25 [M-H]^−^ (C_21_H_20_O_12_) was led to the identification of quercetin-3-O-glucoside which was further collaborated by comparison of MS/MS fragmentation of the precursor ion at *m/z* 301.17 resulted due to the loss of [M-H-glucoside]^−^ and at *m/z* 300.17 produced by the elimination of [M-H-glucoside radical]^−^ (Table 3). The fragmentation pattern for quercetin-3-O-glucoside identified in this study is in good agreement with some other studies presented by Falcão et al. [23] and Falcão et al. [24].

Peak at *m/z* 577.33 [M-H]^−^ (C_27_H_30_O_14_) in mass spectrum (Figure 3) was identified as apigenin-O-rutinoside, which was further confirmed by MS/MS fragment ion peaks at *m/z* 559.33 that corresponds to the elimination of [M-H-H_2_O]^−^ and at *m/z* 269.33 due to the elimination of [M-H-rutinoside]^−^ (Table 3).

A precursor ion at *m/z* 609.33 [M-H]^−^ (C_27_H_30_O_15_) in the negative ionization mode of mass spectrum (Figure 3) and its MS/MS spectrum showed fragment ion peak at *m/z* 301.17, characteristic peak of rutin, formed after the loss of hexose residue (162 amu) or the direct loss of rutinoside residue [M-H-rutinoside]^−^. Therefore, these data and comparison with the literature produced by Cuyckens and Claeys, [31] led to the identification of peak as quercetin-3-*O*-rutinoside (rutin). The peak at *m/z* 301 for rutin was also reported by Ibrahim et al. [32].

A peak at *m/z* 623.50 [M-H]^−^ (C_28_H_32_O_16_) in mass spectrum (Figure 3) was identified as isorhamnetin-3-O-rutinoside, which showed MS/MS fragmentation ion peaks at *m/z* 315 by the elimination of [M-H-rutinoside]^−^ and at *m/z* 300.33 by the loss of [M-H-rutinoside-^·^CH_3_]^−^ (Table 3). The fragment ion peaks identified in this study resembled the previous data reported by Falcão et al. [23] and Falcão *et al*. [24].

A peak at *m/z* 637.42 in mass spectrum (Figure 3) was identified as quercetin dimethyl ether-3-O-rutinoside, which showed MS/MS fragment ion peak at *m/z* 301.17 [M-H-2 CH3-rutinoside]^−^ (Table 3). The present finding is in good agreement with the previously described fragment ions of quercetin dimethyl ether-3-O-rutinoside by Falcão et al. [23] and Falcão et al. [24].

**Figure 4:**
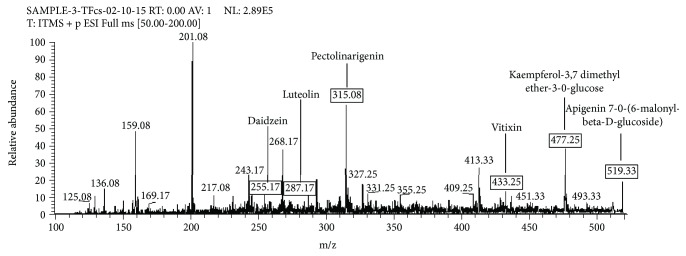
LC-MS/MS spectrum (*m/z* 100–500) of flavonoid fraction of *Coriandrum sativum* generated through positive ionization mode.

Six flavonoids including daidzein, luteolin, pectolinarigenin, apigenin-C-glucoside, kaempferol-3-7-dimethyl ether-3-O-glucoside, and apigenin-7-O-(6-methyl-beta-D-glucoside) were identified in the bioactive fraction of *Coriander sativum* when analyzed through positive ionization mode of LC-ESI-MS/MS. The fragmentation pattern of these flavonoids presented with their MS^2^ spectra was comparable with previously reported literature [33, 34]. Precursor ion peak at *m/z* 255.17 [M + H]^+^ (C_15_H_10_O_4_) in the mass spectrum (Figure 4) was identified as daidzein which produced fragment ion peaks at *m/z* 237.17 that corresponds to the loss of [M + H-OH]^+^ and at *m/z* 227.17 by the loss of [M + H-CO]^+^ in MS/MS spectrum specific for daidzein molecule (Table 4).

Luteolin (C_15_H_10_O_6_) produced precursor ion peak at *m/z* 287.17 [M + H]^+^ in mass spectrum (Figure 4) and further confirmed from MS/MS fragment ion peaks at *m/z* 269.08 after the elimination of [M + H-H_2_O]^+^ and at *m/z* 259.17 after the removal of [M + H-CO]^+^ (Table 4). The fragment ion peaks were in agreement with the fragment ion peaks of luteolin described by Santos et al. [34] and Ibrahim et al. [32].

The precursor ion peak at *m/z* 315.08 [M + H]^+^ (C_17_H_14_O_6_) in mass spectrum (Figure 4) was detected as pectolinarigenin. This peak was further confirmed through MS/MS fragment ion peaks produced at *m/z* 297.17 by the loss of [M + H-H_2_O]^+^ and at *m/z* 243.08 by the elimination of [M + H − C_3_H_4_O_2_]^+^ (Table 4).

A precursor ion at *m/z* 433.25 [M + H]^+^ (C_21_H_20_O_10_) was identified as apigenin-8-C-glycoside (apigenin-C-glucoside) in mass spectrum (Figure 4). The main product ion peaks in the MS/MS spectrum during positive ionization mode produced at *m/z* 415.25 due to dehydrations [M + H − H_2_O]^+^ and at *m/z* 271.08 due to the cleavage of the sugar ring [M + H-glucoside]. These product ion peaks were also resembled to the peaks of apigenin-C-glucoside described in another study by Abad-Garcia et al. [33] (Table 4). The peak at *m/z* 477.25 [M + H]^+^ (C_23_H_24_O_11_) corresponds to kaempferol 3,7-dimethyl ether-3-O-glucoside in mass spectrum (Figure 4), which was further confirmed through the fragment ion peaks produced at *m/z* 315.17 by the loss of glucoside unit [M + H − glucoside]^+^ in the MS/MS spectrum (Table 4). A molecular ion peak at *m/z* 519.33 [M + H]^+^ (C_24_H_22_O_13_) was identified as apigenin-7-O-(6-malonyl-beta-d-glucoside) in mass spectrum (Figure 4), which produced fragment ion peaks at *m/z* 271.08 after the removal of [M + H-malonyl-glucoside]^+^ in the MS/MS spectrum (Table 4).

## 4. Discussion

In the present study, four secondary metabolites including flavonoids, tannins, alkaloids, and steroids were fractionated from *Coriandrum sativum* and evaluated for ACE inhibition potential to find out the actual bioactive compounds responsible for ACE inhibition. Extraction and isolation of desired components from the plants is not only necessary for purification and characterization purpose but also important for the identification of particular bioactive compounds used as ACE inhibitors. The ACE inhibition potential of the flavonoid-rich fraction of *Coriandrum sativum* was higher than all other studied fractions. Higher ACE inhibition potential of only flavonoid might be due to the presence of ACE inhibitor bioactive compounds in this fraction. The leaves of *Coriandrum sativum* possess a significant ACE inhibition potential, while the seeds of this plant did not show any ACE inhibition potential [8]. The high ACE inhibition potential of fresh leaves than the seeds of *Coriandrum sativum* is because of variation in nature and concentration of bioactive compounds in different parts of the same plant. Moreover, the frequency distribution of variety of phytoconstituents in various parts of the plants may be different due to species and geonetical variations [35–37].

Peptides and flavonoids are the most investigated classes of phytochemicals for their ACE inhibition potential. Plant peptides commonly have IC_50_ values in the range of 16–310 *μ*g/mL [28, 38, 39]. The flavonoid fraction extracted from *Coriandrum sativum* showed IC_50_ value 28.91 *μ*g/mL which confirmed that ACE inhibition potential of flavonoid fraction of *Coriandrum sativum* is comparable to ACE inhibition potential of peptides of other plants.

Flavonoids are considered as chief antioxidants in the medicinal plants and can play a pivotal role in the prevention of cardiovascular and other oxidative stress-related disorders. Other than combating free radicals, flavonoids are also having antihypertensive, antihistamine, antimicrobial, memory enhancing, and even mood-boosting properties. A large number of experimental studies have been available to prove the use of flavonoid-rich food, supplements, or herbal preparation for the protection and treatment of atherosclerosis and cardiovascular diseases [29, 40–44]. Natural flavonoids, including hesperidin, rutin, and diosmetin, are utilized as basic ingredient in more than hundred herbal medicines that are being sold throughout the world [45]. Remedial properties of flavonoids have been attributed to their ability to act as antioxidants, free radical scavengers, and chelators of divalent cations. The biological mechanisms of flavonoids to control vascular function and hypertension seem to be concomitant with the accomplishment of nitric oxide (NO). Although the mechanism involved in increased in production of NO is not fully understood, but it is expected that NO production might be controlled by the regulation of the renin angiotensin aldosterone system in endothelial cells. ACE, being a key regulator of the renin-angiotensin-aldosterone system (RAAS), is if inhibited with flavonoids, can easily manage the blood pressure and related cardiovascular disorders. ACE has been reported for exhibiting three active sites: a zinc ion, carboxylate-binding functionality, and a pocket that chelates a hydrophobic side chain of C-terminal amino acid residues. ACE binds to the substrate through coordination with zinc ion present in the structure of the substrate; as a result of this binding, the carbonyl group becomes polarized and facilitate the nucleophilic attack. Therefore, some flavonoids were reported to show *in vitro* ACE inhibition activity through chelation with zinc ion present on the active site of ACE. Free hydroxyl groups of flavonoids have also been reported for the formation of chelate with zinc ions present on ACE [46]. Possibly, flavonoids have functional groups which are able to form hydrogen bridges with the amino acids near at the active site [47–49].

Quercetin, rutin, apigenin, and luteolin have already been identified in *Coriandrum sativum* [50–52], and many others have been identified through this study. Furthermore, the leaves of *Coriandrum sativum* as a source of important therapeutical flavonoids particularly with ACE inhibition mechanism to combat with various cardiovascular diseases were explored for the first time in this study.

**Scheme 1:**
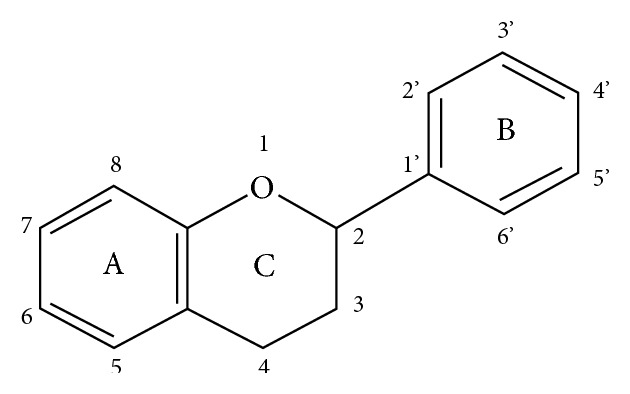


The ACE inhibition potential of different flavonoids is based on three principle structural features which include the presence of the double bond between C2 and C3 at C-ring, the presence of OH group at position 3′, 4′ of B ring [53, 54], and the carbonyl (CO) group on the C4 carbon in the C-ring [55] (Scheme 1).

The presence of a double bond between C2 and C3 is essential to maintain the planner structure of flavonoids responsible for inhibition of ACE. The presence of the different hydroxyl groups also has prominent importance for the establishment of ACE inhibition potential of flavonoids [53]. The exact position and number of the hydroxyl and carbonyl functional groups are key factors for ACE inhibition potential [56, 57]. All the identified flavonoids of *Coriandrum sativum* possess the essential structural features required for the activity to regulate the ACE to ultimate control and manage blood pressure and other related cardiovascular diseases.

Some of the flavonoid including pinocembrin, apigenin, quercetin, and rutin have already been reported as ACE inhibitors [5, 58], while many other important flavonoids were explored in this study including pseudobaptigenin, galangin-5-methyl ether, baicalein trimethyl ether, kaempferol dimethyl ether, pinobanksin-5-methylether-3-O-acetate, pinobanksin-3-O-pentenoate, pinobanksin-3-O-phenylpropionate, pinobanksin-3-O-pentanoate, apigenin-7-O-glucuronoide, quercetin-3-O-glucoside, apigenin-3-O-rutinoside, isorhamnetin-3-O-rutinoside, quercetin dimethyl ether-3-O-rutinoside, daidzein, luteolin, pectolinarigenin, apigenin-C-glucoside, kaempferol-3-7-dimethyl ether-3-O-glucoside, and apigenin-7-O-(6-methyl-beta-D-glucoside) that have never been evidenced earlier as ACE inhibitors. All the flavonoids identified in *Coriandrum sativum* except pinocembrin, pinobanksin-5-methylether-3-O-acetate, pinobanksin-3-O-pentenoate, pinobanksin-3-O-phenylpropionate, and pinobanksin-3-O-pentanoate exhibited key structural features required for structural activity relationship to act as ACE inhibitors [58].

## 5. Conclusion

In addition to some already reporting flavonoids, many other new flavonoids like pseudobaptigenin, galangin-5-methyl ether, baicalein trimethyl ether, kaempferol dimethyl ether, apigenin-7-O-glucuronoide, quercetin-3-O-glucoside, apigenin-3-O-rutinoside, isorhamnetin-3-O-rutinoside, quercetin dimethyl ether-3-O-rutinoside, daidzein, luteolin, pectolinarigenin, apigenin-C-glucoside, kaempferol-3-7-dimethyl ether-3-O-glucoside, and apigenin-7-O-(6-methyl-beta-D-glucoside) identified in the leaves of *Coriandrum sativum* exhibited such structural features which are essential to inhibit the activity of angiotensin-converting enzyme. Therefore, the leaves of *Coriandrum sativum*, as a source of therapeutic flavonoids, can manage blood pressure very efficiently. *Coriandrum sativum*, a common functional food, if used in an appropriate way, can prevent and even combat a variety of cardiovascular disorders.

## Figures and Tables

**Table 1:** ACE inhibition potential of bioactive compounds extracted from *Coriandrum sativum*.

Treatments (100 *μ*g/mL)	ACE inhibition (%)
Steroids fraction	−0.9 ± 1.47C
Alkaloids fraction	−32.79 ± 1.97D
Flavonoids fraction	81.4 ± 0.48A
Tannins fraction	2.3 ± 0.64B

Means sharing different letter are statistically significant (*P* > 0.05).

**Table 2:** IC_50_ values of flavonoid fraction of *Coriandrum sativum* and captopril.

Sr. no.	Extracts	IC_50_ (*μ*g/mL)
1	Flavonoid fraction	28.91 ± 13.42
2	Captopril	4.68 ± 15.42

**Table 3:** Name and structure of flavonoids identified from the flavonoid fraction of *Coriandrum sativum* through LC-MS in negative ionization mode.

Sr. no.	Name of compounds	MW	R_t(Min)_	*λ *_max_ (nm)	HPLC/ESI-MS *m/z* [M-H]^−^	HPLC/ESI-MS/MS *m/z* [M-H]^−^
1	Pinocembrin	256.25	6.20	288	255.25	237.17, 213.17, 211.17
2	Apigenin	270.24	7.39	325	269.33	153.08
3	Pseudobaptigenin	282.24	7.56	**—**	281.25	263.25, 237.17
4	Glangin-5-methyl ether	284.27	7.60	259,350	283.33	268.08, 239.25, 211.17
5	Quercetin	302.24	7.69	256	301.33	273.25, 257.25, 151.17
6	Baicalein trimethyl ether	312.32	7.75	**—**	311.17	257.17, 249.25, 153
7	Kaempferol dimethyl ether	314.29	7.79	339	313.75	298.08
8	Pinobanksin-5-methyl ether-3-O-acetate	328	7.84	292	327.33	285.33, 267.08, 239.05
9	Pinobanksin-3-O-pentenoate	354.11	7.98	291	353.25	271.33, 253.08
10	Pinobanksin-3-O-pentanoate	356.11	8.02	292	355.42	271.33, 253.08
11	Pinobanksin-3-O-phenyl propionate	404.25	8.36	292	403.25	325.17, 253.17
12	Apigenin-7-O-glucuronide	446	8.42	325	445.17	269.08, 175.17
13	Quercetin-3-O-glucoside	464	8.49	256,354	463.17	301.17, 300.17
14	Apigenin-3-O-rutinoside	578	8.96	325	577.33	559.33, 269.33
15	Rutin	612	9.29	256,353	611.40	609.25, 301.17
16	Isorhamnetin-3-O-rutinoside	624	9.46	253,346	623.50	315.00, 300.33
17	Quercetin dimethyl ether-3-O-rutinoside	638	9.60	253,349	637.25	301.17

**Table 4:** Name and structure of flavonoids identified from the flavonoid fraction of *Coriandrum sativum* through LC-MS in positive ionization mode.

Sr. no.	Name of compounds	MW	R_t(Min)_	*λ *_max_ (nm)	HPLC/ESI-MS *m/z* [M-H]^−^	HPLC/ESI-MS/MS *m/z* [M-H]^−^
1	Daidzein	254	0.75	**—**	255.17	237.17, 227.17
2	Luteolin	286	0.91	253,350	287.17	153.08
3	Pectolinarigenin	314	1.23	**—**	315.08	297.17, 243.08
4	Apigenin-C-glucoside	432	1.72	268, 325	433.25	415.25, 271.08
5	Kaempferol-3,7-dimethyl ether-3-O-glucoside	476	1.98	265,346	477.17	315.17
6	Apigenin 7-O-(6-malonyl-beta-d-glucoside)	518	2.39	325	519.33	271.08

## Data Availability

The data in the form of figures/tables and MS spectrum used to support the findings of this study are included within the article.
